# Trap Nesting Wasps and Bees in Agriculture: A Comparison of Sown Wildflower and Fallow Plots in Florida

**DOI:** 10.3390/insects8040107

**Published:** 2017-10-10

**Authors:** Joshua W. Campbell, Cherice Smithers, Allyn Irvin, Chase B. Kimmel, Cory Stanley-Stahr, Jaret C. Daniels, James D. Ellis

**Affiliations:** 1 Steinmetz Hall, Department of Entomology and Nematology, University of Florida, Natural Area Dr., Gainesville, FL 32611, USA; joshuacampbell@ufl.edu (J.W.C.); allynirvin@gmail.com (A.I.); cbkimmel@ufl.edu (C.B.K.); jdaniels@flmnh.ufl.edu (J.C.D.); jdellis@ufl.edu (J.D.E.); 2 Upland Habitat Research & Monitoring, Wildlife Research Laboratory, Fish and Wildlife Research Institute (FWRI), 1105 SW Williston Road, Gainesville, FL 32601, USA; 3 Wildlife International, Progress Park, Alachua, FL 32615, USA; 4 McGuire Center for Lepidoptera and Biodiversity, Florida Museum of Natural History, 3215 Hull Road, P.O. Box 112710, Gainesville, FL 32611-2710, USA

**Keywords:** trap-nest, wildflower plots, *Pachodynerus*, *Euodynerus*, *Isodontia*, *Megachile*

## Abstract

Wildflower strip plantings in intensive agricultural systems have become a widespread tool for promoting pollination services and biological conservation because of their use by wasps and bees. Many of the trap-nesting wasps are important predators of common crop pests, and cavity-nesting bees that utilize trap-nests are important pollinators for native plants and many crops. The impact of wildflower strips on the nesting frequency of trap-nesting wasps or bees within localized areas has not been thoroughly investigated. Trap-nests made of bamboo reeds (*Bambusa* sp.) were placed adjacent to eight 0.1 ha wildflower plots and paired fallow areas (control plots) to determine if wildflower strips encourage the nesting of wasps and bees. From August 2014 to November 2015, occupied reeds were gathered and adults were collected as they emerged from the trap-nests. Treatment (wildflower or fallow plots) did not impact the number of occupied reeds or species richness of trap-nesting wasps using the occupied reeds. The wasps *Pachodynerus erynnis*, *Euodynerus megaera*, *Parancistrocerus pedestris*, and *Isodontia* spp. were the most common trap-nesting species collected. Less than 2% of the occupied reeds contained bees, and all were from the genus *Megachile*. The nesting wasp and bee species demonstrated preferences for reeds with certain inside diameters (IDs). The narrow range of ID preferences exhibited by each bee/wasp may provide opportunities to take advantage of their natural histories for biological control and/or pollination purposes.

## 1. Introduction

The abundance of many bee and other pollinator species has been declining for a variety of reasons, including increased pesticide usage, agricultural practices, habitat fragmentation, invasive species, spread of pathogens, and climate change [1]. Conversely, solitary wasps that may be important for control of pest insects [2] may also be negatively affected by intensive agriculture [3] and, thus, habitat diversity may benefit some predatory wasp species [4]. Many common agricultural practices (e.g., plowing, planting monocultures, etc.) are considered harmful to bees and wasps [5], as well as overall floral and faunal biodiversity [6]. Agricultural intensification has removed pollinator foraging and nesting habitats, resulting in reduced pollination services available for crops [7], and has caused a significant decline of biodiversity [8]. In Europe, sown wildflower strips planted near intensively farmed areas are becoming increasingly popular for the purpose of augmenting insect pollinator populations and biological control agents [9]. Overall, wildflower strips generally positively impact pollinator abundance and species richness [8]. Some researchers have documented higher abundances of pollinators in crops that have wildflower strips growing nearby [10]. However, others have found the opposite for some insect species. For example, despite higher abundances in sown wildflower strips, butterfly species richness was not significantly different in wildflower strips compared to that in extensively managed meadows [11]. There is also some concern that field margins may shelter potential agricultural pests that could spill over onto adjacent crops [12]. Some predatory wasps have been shown to be more abundant in agricultural areas that have greater habitat diversity [4] and, therefore, some agricultural practices could help with pest management. As a result, it is important to know more about how wildflower plantings impact beneficial (pollinators, biological control agents) and pest insect species.

Bees and wasps are examples of beneficial insects that might be impacted by wildflower plantings [8]. Some solitary bees and wasps utilize tunnels in dead wood or grass stems as nest sites (these usually are called “trap-nesting” bees and wasps) [13]. They comprise about 5% of all bee and wasp species [13], and knowing how they are impacted by wildflower plantings is important for a few different reasons. First, these bees and wasps can be used as bioindicators for ecological change [14], with trap-nesting Hymenoptera exhibiting species richness declines within fragmented landscapes [15]. Second, trap-nesting wasp diversity can be an indicator of predator/prey interactions, as they may act as biological controls for many insects [15]. Third, declines in bee diversity or insect pollinator activity have been shown to cause reduced pollen loads on plants, resulting in lower seed set in plants and decreased plant fitness [16,17]. These disruptions of pollination systems can lead to a cascade of bottom-up or top-down ecosystem effects [16].

The aim of this study was to determine if small (0.1 ha) planted wildflower strips can increase the number of bamboo reeds occupied by trap-nesting bees and wasps at a given site. Reeds similar to the ones we used are commonly deployed by homeowners and others to increase nesting sites for bees and wasps that can act as pollinators and/or biological control agents [18]. Graham [19] used trap-nests within the same region as our study, and captured higher abundances of wasps compared to bees despite numerous trap-nesting bees inhabiting this region. However, these trap-nests were randomly placed, and were not placed near wildflowers [19]. We hypothesized that the increased floral resources and potential increased prey abundances provided by planted wildflowers plots would result in increased occupation of reeds by trap nesting bees and wasps near the wildflower plots compared to those in fallow controls.

## 2. Experimental Section

### 2.1. Study Sites

**Figure 1:**
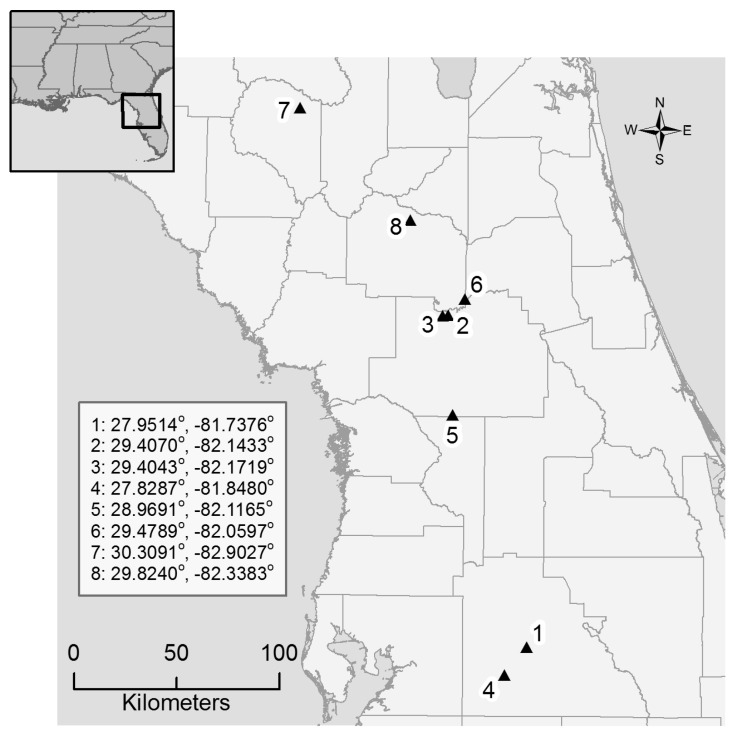
Map depicting locations of the eight sites that included a 0.1 ha wildflower plot and a fallow control plot (the latter a minimum of 500 m away from the wildflower plot). The listed coordinates for each site fall between the wildflower plot and the fallow control plot at the given site. All sites were a minimum of 3 km apart.

Eight sites located within agriculturally dominated areas were selected within North-Central Florida (Figure 1). All of the sites were located near various crop fields. Each site contained two plots (0.1 ha each), one planted with wildflowers and a second as a fallow control plot, that were located approximately 500 m apart. The relatively short distance between the wildflower and control plots ensured that land use would be similar at a given site and that bees and wasps could potentially visit either plot. Each wildflower plot was prepared for seeding by application of glyphosate and mowing to minimize weed competition with the wildflowers. In October/November 2014, nine native annual and perennial herbaceous flowering plant species (Table 1) that had been shown to perform well in north Florida agricultural systems [20] were hand broadcast within the wildflower plot. In the wild, these wildflowers produce and drop most of their seeds in the fall and our seeding attempted to mimic the natural systems. For each wildflower plot, partridge pea was restricted to a small portion of the plot (~15% of the area) due to its potential to outcompete other seeded species. A hand seed roller was used immediately after seeding to maximize seed-to-soil contact. During the following spring and summer, the removal of non-desirable weedy species was performed as needed through hand removal, the application of a post-emergence herbicide (i.e., glyphosate or clethodim), and/or mowing. The unenhanced fallow control plots consisted primarily of grasses (Poaceae or Gramineae). The fallow control plots were similar in plant composition to that of the wildflower plots prior to preparing the plots for seeding. The control plots were maintained at the discretion of the landowner and therefore some were mowed every few weeks, while others were mowed only once or twice a year. No flowering forbs or other plants were documented within the fallow control sites during the course of this research.

### 2.2. Trap-Nests

**Figure 2:**
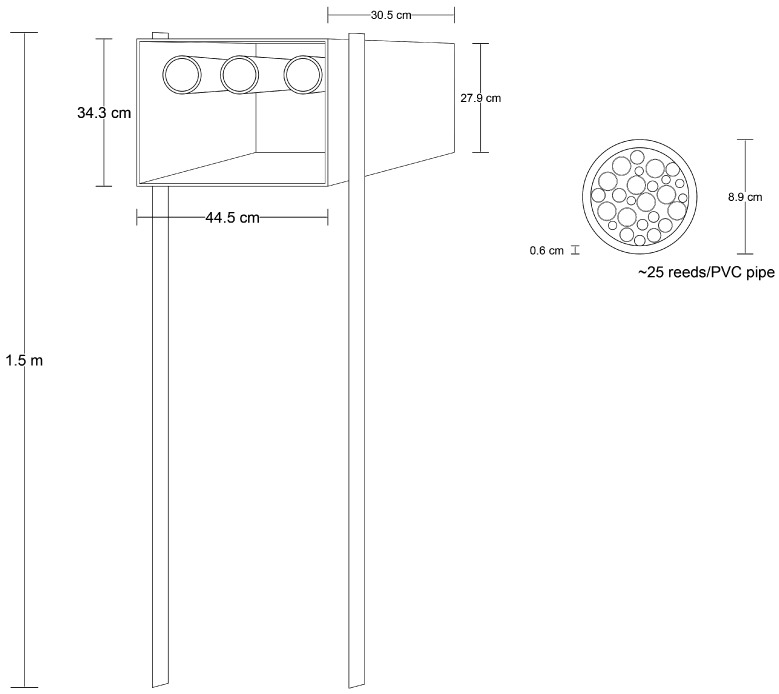
Drawing and measurements of the trap-nest used in this study to collect trap-nesting wasps and bees between August 2014 and November 2015.

A set of trap-nests was placed on the edge of each wildflower and fallow control plot shortly before seeding of the wildflower plots (August 2014). The set of trap-nests consisted of three PVC pipes (ID 8.9 cm) within a corrugated plastic box placed 1.5 m above the ground (Figure 2). Each PVC pipe contained ~25 pieces of bamboo reed (*Bambusa* sp.). Each reed was 19–22 cm in length, and was cut after an internode, or had one side sealed with concrete to ensure a single entrance into the reed. Reeds with openings of varying inside diameters (IDs) were placed within each PVC pipe to provide nesting resources appropriate to accommodate a wide range of potential hymenopteran species. Available reed IDs ranged from 2.8 mm to 13.4 mm. Between August 2014 and November 2015, reeds were checked every 2–3 weeks, and occupied nests (reeds in which nests were constructed) were removed from the nest-box and replaced with new reeds. The occupied reeds were labeled (date collected and site locality), and a plastic vial was placed over the opening of each occupied reed to collect emerging adults. The occupied reeds were stored outside in an open-air enclosure. The ID of each reed opening was measured using a digital caliper after all insects had emerged from the reed. The first insects emerged in October 2014, and the last emerged in May 2016.

### 2.3. Data Analysis

Paired *t*-tests were used to determine differences in the number of (1) nests occupied by the various wasp species (abundance); (2) species within the occupied nests (species richness); and (3) wasp prey item availability between augmented wildflower plots and fallow control plots. Reeds that were occupied between April 2015 and November 2015 were used for this analysis because this was when the wildflower plots were in bloom, and thus when they were able to have the greatest impact on nest construction in the reeds. Reed data were averaged from each site during the collection period and a square root transformation was accomplished to assure normality. Only wasp genera for which a minimum of 25 nested reeds were used were included in the abundance analysis. Bee usage of the reeds never reached this threshold. Correspondingly, bee abundance data were not analyzed. Data transformation did not normalize reed ID data and, therefore, a Kruskal-Wallis test was used to determine how reed ID influenced nest construction by the various wasp and bee genera. All analyses were conducted using Statistix 9.0 (Analytical Software, Tallahassee, FL, USA).

## 3. Results

During the flowering period (April–November 2015), bees and wasps constructed nests in a total of 400 reeds throughout the wildflower and fallow control plots. These occupied reeds yielded 1100 individual Hymenopterans and some associated brood parasites. An additional 286 reeds (475 emerging individuals) were gathered prior to the flowering period (August 2014–March 2015). A total of sixteen species of bees and wasps emerged from the reeds along with 12 natural enemies (other wasps, flies, and beetles) (Table 2). *Pachodynerus erynnis* (Vespidae) was the most common species to emerge from the reeds, emerging from 24.6% of all occupied reeds (Table 2). They were followed by *Euodynerus megaera* (Vespidae, 15% of occupied reeds), and *Parancistrocerus pedestris* (Vespidae, ~12% of occupied reeds) (Table 2). The most common brood parasites were from the genus *Chrysis* (Chrysididae) and were found in 9% of all occupied reeds followed by dipterans (2.8%, fly) and natural enemies Ripiphoridae (2.5%, beetle) (Table 2). Bees from four *Megachile* species emerged from only 11 reeds (1.6% of all occupied reeds) (Table 2). Overall, diverse food types were represented among the trap-nesting wasps/bees [13,21,22] (Table 2) as were variable emerging success rates for the various genera/species (Table 3).

**Figure 3:**
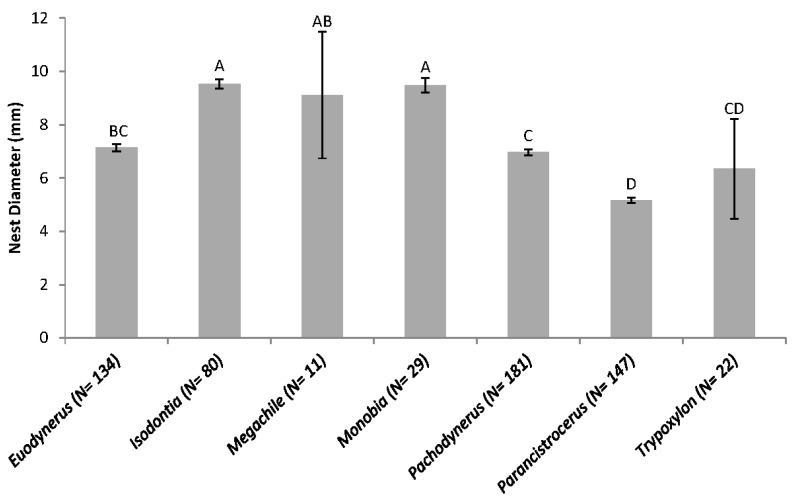
Common genera of wasps and bees and the mean nest entrance inside diameter (ID) (mm ± SE). Sample size (N) is the number of reeds in which nests were constructed and not the number of hatched individuals. The ID range of available reeds was 2.7 mm–13.4 mm. Columns with different letters are significantly different from one another (χ^2^ = 288.76, df = 6, *p* < 0.00001).

No significant differences in the number of occupied reeds by common (N > 25 occupied reeds) wasp genera or number of reeds utilized by all parasites were detected between trap-nests placed adjacent to wildflower and those placed adjacent to fallow control plots (*p* > 0.05; Table 4). Likewise, no significant difference in species richness of bees or wasps in the occupied reeds was detected between the wildflower and fallow control plots (*p* = 0.389). Reed ID significantly impacted which bee and wasp genera would utilize a given reed as a nest site, with many wasp genera preferring to nest within a narrow range of IDs (Figure 3). Wasps or bees emerged from 54% of occupied reeds that also contained natural enemies.

## 4. Discussion

Although wildflower enhancements on farmlands have been used to help augment the numbers of pollinating insects [9,10,23] and arthropod predators [24,25] adjacent to crops, our data indicated that such enhancements did not increase nest construction by trap-nesting bees or wasps. Tscharntke et al. [14] found that trap-nesting wasp occupancy of nests was doubled by adding numerous trap nests, supporting the notion that nest site availability may be more important to nest-seeking wasps than habitat quality.

Tunnel-nesting wasps occupied the majority (~97.5% nests) of reeds containing nests in this study. Only a few megachilid bees utilized the trap-nests (~2.5% nests) (excluding bee/wasp parasites). Similarly, Graham [19] found that a higher percentage of wasps nested in hollow reeds than did bees in the same region our study was conducted. The wasps collected often utilize nectar, and sometimes use pollen as adults [26], but feed their young solely arthropod prey. Therefore, wasps that utilize trap-nests may be more apt at successfully identifying habitats with abundant prey rather than those with high floral diversity [15,27]. Species richness and abundance of trap-nesting wasps have been found to be unrelated to plant species richness [15,27]. Due to the small size of the enhanced wildflower plots utilized in this study, wasps would have foraged outside the experimental plots, thus making it possible that wasp prey could also have gathered outside the experimental plots. Pfiffner et al. [28] found that enhanced wildflower strips did not always increase lepidopteran egg predator or parasite abundance and that their abundance was more closely related to site-specific environmental factors. Trap-nests have been used as a conservation tool to augment pollinating bees numbers, but have also been found to be attractive to wasps that can contribute to pollination and also help to control some arthropod pest species [18].

Bee/wasp utilization of reeds with openings of various IDs was significantly different among the genera, such that genera with larger/longer bodies generally nested in reeds with larger ID openings. Wasps and bees have been shown to have nest entrance size preferences [14,25,29]. Several cavity-nesting Megachilidae have specific nest opening size and structure requirements, and have been managed successfully for pollination services [30,31]. Commercial trap-nests have been designed to trap and maintain populations of many species of Megachilidae bees for crop pollination. Although inundative release of small parasitoid wasps is a common practice to combat some pests [32], no commercial trap-nests for wasps are designed and utilized for biological control. The majority of the aculeate wasp species collected in this study capture microlepidoptera to feed to their young, whereas others prey upon Gryllidae, other Orthopterans, and spiders. Many of these arthropods are common pests within agricultural and residential settings. Due to the narrow range in nest ID preferences of the wasps collected in this study, and their propensity to prey upon certain crop pests, the potential for directed biological control could exist and be augmented using appropriately sized nests.

Megachilidae, the only family of bees using the trap nests in this study, are dependent on nectar as adults, but also gather pollen and some nectar for the mass provisioning of their young. Interestingly, *Megachile lanata* was found in our southernmost experimental site. Native to India and parts of North Africa, it spread to the Antilles during the slave trade [33], and later expanded to southern Florida. To our knowledge, this is the first record of this bee collected from a trap-nest. In India, it is an important pollinator of sunn hemp (*Crotalaria juncea*), which is being experimentally tested as a cover crop in Florida [34]. Sunn hemp cannot be pollinated by small bees, and a lack of appropriate pollinators can result in reduced seed set [34]. Therefore, *M. lanata* could be used as a pollinator of sunn hemp if this plant becomes commercially grown in Florida.

Species richness and abundance of trap-nesting bees and wasps appeared to vary greatly among the sites, suggesting that many of these species have patchy distributions. This variation could be due to many factors, such as nesting/floral resource availability in time and space, pesticide use, surrounding land-use, or other site-specific characteristics [35]. The nest IDs of the majority of wasps have not been documented and our data suggests that some wasp species may be attracted to certain nest IDs.

The potential agricultural utility of wildflower strips should not be underestimated, as many benefits have been documented [9]. Habitat patches left within cropped areas of large-scale agricultural systems can increase native bee abundance and the corresponding pollination services they provide to adjacent crops [3,36]. However, whether these beneficial species are simply foragers, or are utilizing the nearby patches for nesting sites is poorly understood. Floral resources are important for sustaining and attracting foraging pollinators. Despite this, nesting structure needs have largely been ignored by most researchers, and should be a priority for bee conservation [37]. Our data support the idea that nesting material can be used by trap-nesting wasps, regardless of the surrounding vegetation.

## Figures and Tables

**Table 1:** List of native wildflowers and seed rates used to establish the wildflower plots for this study. * Partridge pea was planted separately, i.e., in its own strip that occupied ~15% of the wildflower plot. The seeds were purchased from Wildflower Seed and Plant Growers Association (Crescent City, FL, USA).

Plant List	Type	Seed Rate (kg/ha)
Partridge Pea (*Chamaecrista fasciculata*)	Annual	1.12 *
Goldenmane Tickseed (*Coreopsis basalis*)	Annual	1.01
Lanceleaf Tickseed (*Coreopsis lanceolata*)	Perennial	0.19
Leavenworth's Coreopsis (*Coreopsis leavenworthii*)	Perennial	0.19
Indian Blanket (*Gaillardia pulchella*)	Annual	3.03
Swamp Sunflower (*Helianthus angustifolius*)	Perennial	0.37
Spotted Beebalm (*Monarda punctata*)	Perennial	0.15
Blackeyed Susan (*Rudbeckia hirta*)	Annual	0.59
Tall Ironweed (*Vernonia angustifolia*)	Perennial	0.69

**Table 2:** List of all insect species and number of reeds occupied by the species in the trap-nests from wildflower and fallow control plots at eight sites in Florida.

Order	Family	Species	Site 1	Site 2	Site 3	Site 4	Site 5	Site 6	Site 7	Site 8	Total	Food/Food Breadth
Hymenoptera (Wasps)	Chrysididae	*C. inaequidens/wasbaueri*	0	1	0	1	0	0	0	1	3	Wasp/Bee
		*Chrysis inaequidens*	0	1	0	4	5	3	5	1	19	Wasp/Bee
		*Chrysis nisseri*	0	0	0	0	0	3	1	3	7	Wasp/Bee
		*Chrysis pellucidula/remissa*	0	0	0	0	0	2	1	0	3	Wasp/Bee
		*Chrysis remissa*	0	4	0	0	0	14	1	0	19	Wasp/Bee
		*Chrysis tripartita*	0	0	0	0	0	1	0	0	1	Wasp/Bee
		*Chrysis wasbaueri*	0	3	0	1	0	1	1	4	10	Wasp/Bee
	Leucospididae	*Leucospis affinis*	0	0	0	0	0	0	0	1	1	Bee
	Crabronidae	*Trypoxylon collinum*	0	8	1	0	8	1	0	1	19	Araneae
		*Trypoxylon lacitarse*	0	0	0	0	0	0	2	1	3	Araneae
	Sphecidae	*Isodontia auripes*	0	14	14	1	6	0	0	9	44	Orthoptera
		*Isodontia Mexicana*	1	15	2	0	2	0	14	2	36	Orthoptera
	Vespidae	*Euodynerus annulatus*	0	0	0	4	1	0	1	0	6	Lepidoptera
		*Euodynerus hildalgo*	0	8	0	3	0	3	2	0	16	Lepidoptera
		*Euodynerus megaera*	0	6	0	0	30	18	13	36	103	Lepidoptera
		*Monobia quadridens*	0	11	0	0	3	3	0	12	29	Lepidoptera
		*Pachodynerus erynnis*	2	36	22	24	5	40	37	3	169	Lepidoptera
		*Pachodynerus nasidens*	0	0	0	7	0	0	0	0	7	Lepidoptera
		*Parancistrocerus pedestris*	3	29	7	0	1	5	29	6	80	Lepidoptera
		*Parancistrocerus fulvipes*	0	23	13	0	0	4	14	10	64	Lepidoptera
Hymenoptera (Bees)	Megachilidae	*Megachile lanata*	0	0	0	2	0	0	0	0	2	Polylectic
		*Megachile parallela*	0	0	0	0	0	0	1	0	1	Polylectic
		*Megachile policaris*	0	0	0	5	0	0	0	0	5	Polylectic
		*Megachile xylocopoides*	0	1	0	0	0	0	2	0	3	Polylectic
Diptera	Bombyliidae	*Toxophora amphitea*	1	2	0	1	1	1	3	0	9	Wasp/Bee
	Sarcophagidae	Miltogramminae	1	3	0	1	1	1	2	1	10	Wasp/Bee
		(incl. *Amobia* sp., *Senotainia* sp., *Phrosinella* sp.)										
Coleoptera	Ripiphoridae	*Macrosiagon cruenta*	1	5	1	2	2	1	1	3	16	Wasp
		*Macrosiagon* sp.	0	0	0	0	0	0	0	1	1	Wasp
		Total	9	170	60	56	65	101	130	95	686	

**Table 3:** Average # adults (SE) that emerged from reeds and ranges of adult emergences for common species/genera.

	Avg. #/Reed	Range
*Isodontia auripes*	3.0 (0.3)	1–8
*Isodontia mexicana*	3.7 (0.3)	1–7
*Trypoxylon* spp.	3.9 (0.5)	1–10
*Euodynerus annulatus*	2.2 (0.5)	1–3
*Euodynerus hildalgo*	3.1 (0.7)	1–11
*Euodynerus megaera*	2.0 (0.4)	1–8
*Monobia quadridens*	1.0 (0.3)	1–4
*Pachodynerus erynnis*	2.8 (0.2)	1–7
*Pachodynerus nasidens*	1.0 (0.4)	1–4
*Parancistrocerus pedestris*	1.1 (0.1)	1–6
*Parancistrocerus fulvipes*	1.0 (0.2)	1–3
*Megachile* spp.	2.7 (0.6)	1–6

**Table 4:** Mean (±SE) abundance of occupied nests by the various wasp species, total parasites and species richness of wasps and bees collected April 2015–November 2015 between fallow control plots and augmented wildflower plots.

	Fallow Controls	Wildflower Plots	P_trt_ (df = 7)
*Euodynerus megaera*	1.9 (1.7)	1.6 (1.0)	0.85
*Isodontia auripes*	1.8 (1.6)	2.0 (1.1)	0.56
*Isodontia mexicana*	1.1 (0.7)	3.1 (1.5)	0.08
*Pachodynerus erynnis*	8.0 (3.3)	6.1 (2.3)	0.90
*Parancistrocerus pedestris*	4.6 (2.2)	4.6 (1.9)	0.90
*Parancistrocerus fulvipes*	2.5 (1.0)	1.8 (0.9)	0.55
Total parasites	3.1 (1.5)	2.3 (1.0)	0.66
Species richness	4.4 (0.8)	5.2 (0.7)	0.39
